# Early-Stage Infection-Specific *Heterobasidion annosum* (Fr.) Bref. Transcripts in *H. annosum*–*Pinus sylvestris* L. Pathosystem

**DOI:** 10.3390/ijms252111375

**Published:** 2024-10-23

**Authors:** Maryna Ramanenka, Dainis Edgars Ruņģis, Vilnis Šķipars

**Affiliations:** Latvian State Forest Research Institute “Silava”, 111 Rīgas Street, LV-2169 Salaspils, Latvia

**Keywords:** plant pathology, root rot, pathogen gene expression, early-stage infection

## Abstract

Transcriptomes from stem-inoculated Scots pine saplings were analyzed to identify unique and enriched *H. annosum* transcripts in the early stages of infection. Comparing different time points since inoculation identified 131 differentially expressed *H. annosum* genes with *p*-values of ≤0.01. Our research supports the results of previous studies on the Norway spruce–*Heterobasidion annosum s.l.* pathosystem, indicating the role of carbohydrate and lignin degradation genes in pathogenesis at different time points post-inoculation and the role of lipid metabolism genes (including but not limited to the delta-12 fatty acid desaturase gene previously reported to be an important factor). The results of this study indicate that the malic enzyme could be a potential gene of interest in the context of *H. annosum* virulence. During this study, difficulties related to incomplete reference material of the host plant species and a low proportion of *H. annosum* transcripts in the RNA pool were encountered. In addition, *H. annosum* transcripts are currently not well annotated. Improvements in sequencing technologies (including sequencing depth) or bioinformatics focusing on small subpopulations of RNA would be welcome.

## 1. Introduction

Scots pine (*Pinus sylvestris* L.) is the most widely distributed pine species and is found throughout Eurasia [1]. It is a species of major economic importance, widely used in timber, pulp, and paper production [2]. It is also a keystone species providing stability for large ecosystems [3,4] and has been a dominant species for millennia [5].

One of the main causes of pine mortality, excluding bark beetles (genera *Ips* and *Tomicus* [6,7]), are *Heterobasidion* complex fungi, in particular, the basidiomycete *Heterobasidion annosum* (Fr.) Bref., which causes root rot [8]. In the European forest sector, the *Heterobasidion* complex causes combined economic losses of hundreds of millions of euros annually [9]. While the exact proportion of the losses specifically related to Scots pine is not known, given the widespread distribution of Scots pine in European forests and the high economic value of Scots pine, the economic impact of this pathosystem on Scots pine is significant.

Currently, mitigation of *Heterobasidion* root rot includes forest management activities (monitoring, creation of sustainable forests, sanitary felling, etc.) [10,11,12,13]. One of the strategies for the protection of renewed pine stands is the use of biological control agents based on the fungus *Phlebiopsis gigantea* (Fr.) Jülich [14,15,16]. Breeding for resistance or tolerance is promising, as the genetic components for *H. annosum* resistance have been determined [17], Scots pine clones with varying resistance against *H. annosum* have been described [18], and the heritability and genetic gain values for breeding for resistance against root rot have been calculated [19].

To increase the efficiency of breeding programs, genetic mechanisms and candidate genes for resistance need to be identified; thus, studies on gene expression changes in the host in response to inoculation and related genetic testing have been performed for Scots pine reacting to *H. annosum*, and also for related pathosystems [20,21,22,23,24]. Genetic virulence factors of *H. annosum* have also been studied [25,26]. A study combining genomic and transcriptome approaches identified genes linked with secreted proteins as potential virulence factors (besides genes involved in oxidation–reduction processes and genes encoding domains relevant to transcription factors) [27]. In the same study, secretome annotation and analysis in the pathogen–host interactions database [28] showed that the most virulent *Heterobasidion parviporum* isolate was found to contain many carbohydrate-active enzyme genes for cell wall degradation and an increased amount of secreted proteins for lignin degradation. Proteins of the reactive oxygen species-scavenging system and proteases were identified as an important part of the secretome. This study also identified cytochrome P450 proteins as significant for pathogenesis, which could be explained by the involvement of cytochrome P450 gene family members in the detoxification of substances from the environment and the synthesis of fungal toxins [29]. However, the study by Zeng et al. [27] did not identify a specific gene as the sole determinant for variations in virulence between different *H. parviporum* isolates.

Recently, dual transcriptome studies have provided information on host and pathogen transcriptomes simultaneously [30,31,32,33]. However, not all of these studies focused on the host–pathogen interactions [31]. Dual transcriptome studies are also possible only if a high number of reads can be obtained from samples to provide a sufficient number of pathogen transcriptome reads, as the proportion of transcripts from the pathogen can be below 1% [32]. No dual transcriptome studies on the *H. annosum*–*P. sylvestris* pathosystem have been published and, to the best of our knowledge, there are no investigations providing data on the *H. annosum* (*sensu stricto*) transcriptome during infection of Scots pine. However, in a study by Lunden et al. (2015) about the inoculation of Norway spruce with *H. annosum* s.s., delta-12 fatty acid desaturase and clavaminate synthase were indicated as potential virulence factors [33]. Polysaccharide-degrading enzymes and lignin-degrading enzymes have long been regarded as important for pathogenesis [34]. Expression of these genes was also detected in a dual transcriptome experiment by Kovalchuk et al. (2019) [30]. The same study also mentioned that seven *Heterobasidion* genes classified as lipases were expressed, but no further details were provided in that study. The expression of lipid metabolism genes (encoding lipases and the malic enzyme) was detected in *Fusarium circinatum* Nirenberg & O’Donnell while infecting *Pinus* species [32]. In addition to the expression of polysaccharide- and lignin-degrading enzyme genes, the accumulation of toxins and oxalate has been reported in host tissues colonized by *Heterobasidion* [34].

Previous research has shown that analysis of fungal transcriptomes enables the understanding of adaptations by pathogens to different host species [32]. Therefore, the accumulation of in planta gene expression data for *H. annosum* can enable the analysis of the variation in defense strategies within the host species by inoculation experiments with genetically characterized fungal isolates. This could be used as a basis for the identification of molecular markers for resistance-oriented forest tree breeding. In addition, a comparison of fungal gene expression between isolates could provide insights into differences in aggressiveness or virulence, providing a better understanding of the role of genetic variation of pathogens in the development of plant diseases. This investigation provides an initial assessment of time post-inoculation-dependent differentially expressed pathogen genes from one to four weeks post-inoculation. This assessment is based on mapping a large number of reads to the *H. annosum* transcriptome (>11 million). This provides information about genes and pathways important in early pathogenesis and demonstrates the feasibility of the utilized sequencing technology.

## 2. Results

### 2.1. Sequencing Statistics

From the 16 sequenced libraries, 3.36 × 10^9^ reads were obtained, of which ~11.25 million reads mapped onto the *H. annosum* transcriptome. A summary of the quantity of reads mapping onto the *H. annosum* and *P. sylvestris* transcriptomes from each sequenced library is provided in Table 1.

From material taken one week post-inoculation, 3.84 million *H. annosum* reads were obtained. Material taken at two to four weeks post-inoculation produced, respectively, 2.54, 3.05, and 1.82 million *H. annosum* reads. This shows that a sufficient number of reads for statistical analysis was obtained, regardless of the low RIN values of the samples. Mapping against the transcriptome of *P. sylvestris* produced hits for, on average, 42.51% of the reads, depending on the library.

The high proportion of reads not mapping onto *P. sylvestris* can be explained by the limited nature of the reference transcriptome. A Scots pine reference genome is currently not available, and this influences the annotation and quality control of any Scots pine transcriptome generated before the availability of a high-quality reference genome.

### 2.2. Most Transcribed Genes at Each Time Point

Based on the number of reads mapping onto the *H. annosum* reference transcripts, we determined the 100 most transcribed genes at each time point (Supplementary File S1).

The frequency of level 7 biological process gene ontology (GO) terms was similar between all time points. The unique GO terms for each time point are summarized in Table 2.

Fifty of the most highly expressed genes were shared between all time points, and each time point had fifty unique genes.

### 2.3. Differential Gene Expression

**Figure 1:**
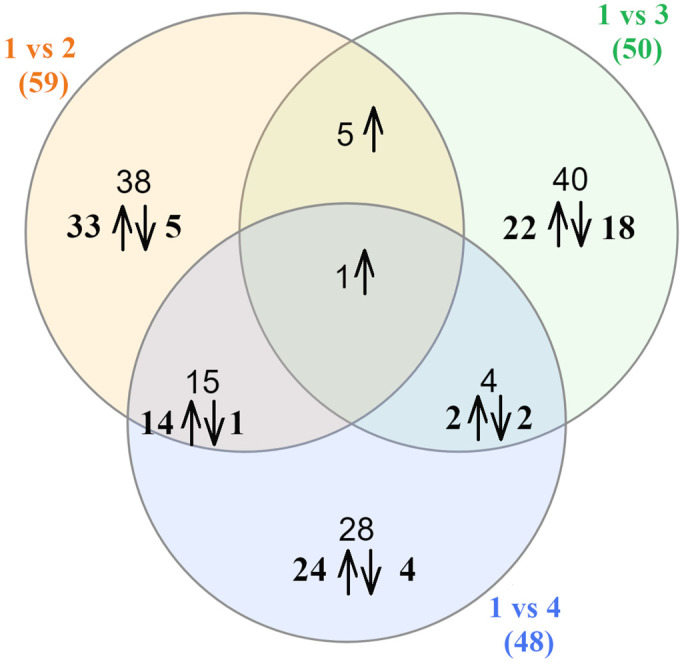
Venn diagram comparing differentially expressed genes in statistical comparisons 1 vs. 2, 1 vs. 3, and 1 vs. 4 WPI. Arrows indicate direction of gene expression regulation.

All time points were compared against all other time points, resulting in six statistical comparisons for differential gene expression (DGE). The main focus of this study was the analysis of early-stage infection transcripts; therefore, for visualizations, the transcriptome one week after inoculation was compared with the other time points (Figure 1). A Venn diagram of the comparison between all time points is provided in Supplementary Figure S1.

The most up- and downregulated transcripts comparing one and two weeks, one and three weeks, and one and four weeks post-inoculation are presented in Table 3, Table 4 and Table 5, respectively. In the Venn diagram above the gene upregulated in all the comparisons is annotated as the malic enzyme.

RT-qPCR validation was not performed on the differentially expressed *H. annosum* genes, as recent research [35,36] indicates that RNA-seq and RT-qPCR results are concordant when gene expression fold changes exceed 2. In this study, the differentially expressed genes listed in Table 3, Table 4 and Table 5 all had fold changes between 8 and 182.

Tables showing the most up- and downregulated transcripts for the rest of the DGE comparisons are provided in Supplementary File S2. A complete expression table is provided in Supplementary File S3.

## 3. Discussion

Differences between the libraries in the proportion of *H. annosum* reads might indicate biologically relevant information, such as the proportion of the living pathogen in the tissue from which RNA was extracted, and other explanations like sampling effects, host genotype effects, unequal rRNA depletion or induced host rRNA production at some time points could influence the results. This could also be an indication of a varying positive effect from the inoculum plug still providing resources to the pathogen, but this hypothesis was not explicitly analyzed in this study. If one outlier (the library with the largest difference from the average percentage of *H. annosum* reads per group) was removed per time point, a strong correlation (R^2^ = 0.8116) between a longer time since inoculation and a lower proportion of *H. annosum* reads was observed. Yet, given the many factors influencing the proportion of *H. annosum* reads, we refrained from conclusions.

Most of the differentially expressed genes lacked detailed annotations; however, some of the genes showing the highest upregulation during infection might have a role in disturbing host cell integrity (e.g., the carotenoid ester lipase precursor, which is secreted and can cross the cell wall [37], and erylysin B [38]), can affect lipid metabolism (e.g., the carotenoid ester lipase precursor, fatty acid desaturase domain-containing protein, delta-12 fatty acid desaturase [33], elongase of fatty acids ELO, and malic enzyme [39]), terpene metabolism (terpenoid cyclases/protein prenyltransferase alpha-alpha toroid), or are involved in oxidation–reduction processes (aldo/keto reductase [40]), cellular growth (GPI mannosyltransferase 3 [41]), cell development and metabolism control (methionine adenosyltransferase (synonym for S-adenosylmethionine synthetase) [42]), and stress tolerance (heat shock protein 70). The increased expression of delta-12 fatty acid desaturases and related protein genes was especially pronounced comparing 1 WPI with 4 WPI. This could be related to lignin degradation [43], especially in the context of elevated transcription of polysaccharide lyase [44]. The initial stage of infection requires the fungal hyphae to penetrate host cell walls to colonize the tissue. As the structural integrity of conifer tissue is mainly ensured by cellulose, hemicellulose, and lignin, polysaccharide-degrading enzymes are needed to penetrate the tissue and access nutrition sources away from potential competitors on the tissue surface. Several microorganisms on soil and tissue surfaces can negatively affect the growth of *H. annosum*; therefore, the ability to quickly penetrate cell walls and colonize tissues is important for the survival of the fungus and successful infection of the host [34].

At one week post-inoculation, a terpenoid cyclase gene was more highly expressed than at two or three weeks post-inoculation. However, as terpenoids represent the most diverse and abundant class of natural products and have high functional diversity [45,46], any suggestions about the possible role in the infection process would be highly speculative.

Genes downregulated during early-stage infection have roles in transcription and translation (transcription regulator, 40S ribosomal protein S26, and RS27A protein), polyamine regulation (ornithine decarboxylase antizyme domain-containing protein [47]), protein folding as chaperones (the groes-like protein and HSP20-like chaperone), and cell development/signaling (cell division control/GTP-binding protein). Decreased activity of the *negative regulator of differentiation 1* gene one week after inoculation could result in the positive regulation of differentiation.

Clearly, improved characterization of the proteins encoded by the differentially expressed genes is needed to obtain a better understanding of the molecular processes affecting *H. annosum*’s pathogenicity. However, the results obtained about the annotated genes suggest that delta-12 fatty acid desaturases could be a virulence factor. Delta-12 fatty acid desaturase gene expression in the *H. annosum*–Norway spruce pathosystem was detected by Lundén et al. (2015) [33]. These authors suggest a central role of this enzyme in sustaining fungal growth. Considering the extremely small amount of *H. annosum* transcripts in the work of Lundén et al. and the small proportion of *H. annosum* reads in our work, the identification of the delta-12 fatty acid desaturase gene as important for the pathogen indicates that this gene plays an important role during infection. An increase in the expression of other transcripts influencing lipid metabolism was also observed (the malic enzyme, elongase of fatty acids, and an unspecified type of fatty acid desaturase domain-containing protein). The gene for the malic enzyme was the only differentially upregulated gene comparing the one-week post-inoculation time point against all other time points. This concurs with findings showing the role of fungal lipids and lipid biosynthesis proteins in plant–pathogen interactions as potential virulence factors [48,49]. The malic enzyme is potentially highly interesting in this context. As reviewed by Voreapreeda et al. (2013) [50], there are different types of malic enzymes (EC 1.1.1.38–EC 1.1.1.40), but the malic enzyme gene identified in our study (sequence ID: CCPC2187.b1 [51]) was not categorized into a specific group. However, the activity of the malic enzyme can drastically change the lipid content of oleaginous fungi [52]. Furthermore, a study investigating the *H. annosum* transcriptome during saprotrophic growth on Scots pine bark, sapwood, and heartwood detected the downregulation of the malic enzyme during growth on bark and heartwood chips [53]. This may suggest that malic enzyme activity is needed specifically when invading a living host; however, further research is needed, as in the previous study, the time post-treatment was 3 months, in contrast with one week in our study.

The observed downregulation of glycoside and glycosyl hydrolase genes at 1 WPI compared with 4 WPI might seem counterintuitive, as enzymes of these groups and carbohydrate-active enzymes, in general, have been recognized as one of the main components facilitating fungal invasion [54,55]. Glycoside and glycosyl hydrolases have a role in the degradation of plant cell wall components; thus, it could be expected to see increased activity of these genes at earlier stages of infection. However, this class of proteins is vast and complex [56], and, therefore, these could be family members with other functions. Members of these groups could be involved in fungal cell wall remodeling, putting it in context with increased fungal growth at later stages of infection.

The upregulation of specific transcription factors sensitive to fungal or plant hormones was not observed, except for one transcription regulator at 1 WPI with limited information about this protein. In general, the biological functions of most highly expressed genes specific to 1 WPI indicate attempts to mitigate oxidative stress and other elements involved in plant defenses. The response to oxidative stress could be linked to protection from reactive oxygen species involved in host defense responses [57]. Alternatively, it could also be associated with the pathogen’s own development-related cell signaling [58] or both. Other biological processes of 1WPI-specific transcripts are related to metabolism, gene expression, and the response to heat, osmotic, and oxidative stress. In addition, the glyoxylate cycle can also facilitate a reduction in oxidative stress [59]. In a broad sense, these transcriptome changes suggest that the pathogen is employing protective measures against plant defenses.

Considering the high proportion of differentially expressed genes lacking detailed annotations, there are still many unknown factors and genes that are involved in the pathogenicity mechanism. More research on the molecular biology of *H. annosum* is needed to gain a better understanding of virulence factors. Further research using genetically identical host plants to obtain a high-quality dual transcriptome of *H. annosum*–*P. sylvestris* interactions at different time points post-inoculation can provide additional information about the interactions between the pathogen and host during the infection process.

## 4. Materials and Methods

One-year-old Scots pine saplings were inoculated with a Latvian isolate (ID HA2) of *H. annosum s.s*. The saplings were obtained from the Latvian State Forests seedling nursery in Kalsnava, Latvia, and were grown from mixed-origin improved seed material. For inoculation, a wooden plug grown with the *H. annosum* isolate was placed on an area of the stem with the bark surface removed. Samples for RNA extraction were collected at one, two, three, and four weeks post-inoculation and stored at −80 °C until RNA extraction. Four biological replicate samples were collected for each time point. RNA extraction was performed following a CTAB-based protocol [60]. The sampling site included the inoculation site. These time points were chosen, firstly, to allow time for the pathogen to grow into the host tissue and form biomass to increase the number of obtainable reads and, secondly, to allow partial comparison with previous studies (Lundén et al., 2015 [33] and Zamora-Ballesteros et al., 2021 [32]), which used 5 and 4 days post-inoculation as their only time points, respectively. In the context of infection progression, in the related *Picea abies* (L.) H. Karst.–*H. parviporum* Niemelä & Korhonen pathosystem, germ tubes form in roots within 24 h after spore adhesion, colonization of cortical tissue happens 24–48 post-inoculation, and the endodermis is reached at 72 h [61]. By 12 to 15 days post-inoculation, stelar cells have deteriorated, but plant responses involving papillae formation and lignification in the cortical and endodermis tissue in the roots can be observed [61]. Thus, the selected time points represent early infection and later stages.

RNA extraction was followed by quality control of the RNA samples using the Agilent 2100 Bioanalyzer and RNA 6000 nano kit (Agilent, Santa Clara, CA, USA, Cat. No. 5067–1511) for RNA integrity determination and quantitation using the Qubit spectrofluorometer and Qubit RNA BR Assay Kit (Thermo Fisher Scientific, Waltham, MA, USA, Cat. No. Q10210). The obtained material was quite degraded. The RIN values ranged from 1.9 to 4.7 (average: 2.6), and three samples did not produce a RIN value but were included as good results and were obtained using qPCR to detect the presence of *H. annosum* RNA in the total RNA samples. Considering that short-read (100 bp) sequencing was used, thus reducing the influence of degraded RNA, sequencing libraries were prepared with all samples. The RNA quality control data are provided in Supplementary Table S1.

To confirm the presence of *H. annosum* RNA in the extracted RNA samples, one-step RT-qPCR with *H. annosum*-specific primers targeting the laccase gene was performed. The GoTaq^®^ 1-Step RT-qPCR system (Promega, Madison, WI, USA, Cat. No. A6021) was used with the forward primer 5′-CCAGAAAGTAGACAATTATTGGATTCG-3′ and reverse primer 5′-GAGTTGCGGCCATTATCGA-3′ [62]. The reaction mixture per sample was 10 µL of GoTaq qPCR Master Mix (2X); 0.4 µL of GoScript RT Mix for 1-Step RT-qPCR (50X); 0.33 µL of CXR reference dye; a final concentration of each primer of 200 nM; and nuclease-free water to a volume of 20 µL. The thermal cycling profile was as follows: 37 °C for 15 min and 95 °C for 10 min, followed by 40 cycles at 95 °C for 10 s, 60 °C for 30 s, 72 °C for 30 s, followed by a melting curve stage, during which the temperature was increased from 60 °C to 95 °C in 0.3 °C intervals. One-step RT-qPCR was performed in an Applied Biosystems StepOnePlus qPCR machine. To avoid the influence of primer dimers on the results, the melting curve peak height for the specific qPCR product was used instead of the Ct value. This approach was possible because none of the reactions reached the plateau phase. The peak height of the specific product was divided by the RNA concentration to obtain a ratio characterizing the proportion of *H. annosum* RNA in the RNA sample.

Transcriptome sequencing libraries were created using the MGIEasy RNA Directional Library Prep Set (MGI Tech Co., Shenzhen, China, Cat. No. 1000006385) following the manufacturer’s protocol. An amount of 500 ng of total RNA was used for each library. Ribosomal RNA was depleted using the Qiagen QIAseq FastSelect–rRNA Plant Kit (Cat. No. 334315). Incubation was performed as described in Table 4 of the QIAseq^®^ FastSelect™ Handbook (according to the treatment of a RIN of <3 samples). The reaction mixture for this step was as follows: 10 µL of the RNA sample containing 500 ng of RNA, 4 µL of fragmentation buffer (MGI), 4 µL of directional RT Buffer 1 (MGI), 1 µL of diluted directional RT buffer 2 (MGI), and 1 µL of QIAseq FastSelect−rRNA plant reagent. RT enzyme mix (MGI) was only added after this incubation, before the reverse transcription step. The rest of the library preparation protocol was not modified from the manufacturer’s protocol. The DNBSEQ-G400RS High-Throughput Sequencing Set (MGI Tech Co., Cat. No. 1000016950) was used for sequencing. Sequencing was performed on a DNBSEQ G400 sequencer (MGI Tech Co.) by the Latvian Biomedical Research and Study Centre (Riga, Latvia) on May 20, 2023. The manufacturer’s standard protocol was used for sequencing.

The CLC Genomics Workbench v.23 was used for data import, and the CLC Genomics Workbench v.21.0.5 (including the Blast2GO commercial plugin v.1.21.14 (BioBam Bioinformatics S.L., Valencia, Spain)) was used for data analysis and annotation. The data analysis workflow was as follows: demultiplexing (automatically performed by the sequencer), trimming (settings: quality limit—0.05; max. number of ambiguous nucleotides—2; no adapter trimming; trim homopolymers; no terminal nucleotide removal; and max. length—150), RNA-seq analysis (settings: no spike-in controls; mismatch cost—2; insertion cost—3; deletion cost—3; length fraction—0.8; similarity fraction—0.8; no global alignment; strand-specific—both; library type—bulk; max. number of hits for a read—10; count paired reads as two—no; expression value—total counts; and create reads track—yes), differential gene expression analysis (settings: technology—whole-transcriptome RNA-Seq; filter on average expression for FDR correction—no; metadata table—yes; test differential expression due to WPI (weeks post-inoculation); controlling for—not set; and comparisons—all group pairs), and the selection of the differentially expressed genes to perform annotation using the Blast2GO plugin (settings: blast program—blastx-fast; Blast DB = nr; and mapping using Goa version 2022_08). The mapping of reads to a reference transcriptome of *H. annosum* was performed [51,63] (settings: masking mode—no masking; update contigs—no; match score—1; mismatch cost—2; cost of insertions and deletions—linear gap cost; insertion cost—3; deletion cost—3; length fraction—0.5; similarity fraction—0.8; no global alignment; auto-detect paired distances; and non-specific match handling—map randomly) to be able to calculate the percentage of *H. annosum* reads. The reads were also mapped against the transcriptome of *P. sylvestris* from Wachowiak et al., 2015 [64] using the same settings.

A gene (transcript) was considered differentially expressed if the comparison between time points produced a statistically significantly different count of reads mapped onto the transcript in question with a *p*-value below 0.01. The results are expressed as fold changes comparing different time points (with four biological replicates for each time point). Calculations of the differential gene expression-related parameters were performed using the differential gene expression analysis tool in the CLC Genomics Workbench software, (see paragraphs 31.6 and 31.6.4 in the manual [65] for more details). Differential gene expression analysis was performed only for *H. annosum* genes.

For the visualization of the differential expression, the online tool for up to 6-group Venn diagram creation InteractiVenn was used [66]. The visualization of the other data was performed using the tools in the CLC Genomics Workbench software v.21.0.5 and the Blast2GO plugin v.1.12.14.

## 5. Conclusions

This study indicates that carbohydrate and lignin degradation gene transcription is essential for the pathogenicity of *H. annosum s.s.* against *P. sylvestris*, similar to the results reported by Kovalchuk et al. [30] in a study on the interaction between Norway spruce and *H. annosum s.l.* In addition, the results from this study suggest that lipid metabolism has a significant role starting from week two post-inoculation. Our data suggest that a number of lipid metabolism pathway genes, in addition to the delta-12 fatty acid desaturase gene, are involved in pathogenicity. Many of the differentially expressed genes were not annotated, and improved annotation of a reference transcriptome of *H. annosum* would provide additional insights into processes occurring during the early stage infection in the *H. annosum*–*P. sylvestris* pathosystem.

## Figures and Tables

**Table 1:** Sequencing statistics: read mapping against *H. annosum* and *P. sylvestris* transcriptomes.

Sample Name ^1^	Total Reads	Percentage of Reads Mapping onto *H. annosum* Transcriptome	Percentage of Reads Mapping onto *P. sylvestris* Transcriptome
1_5	330,514,752	0.32	26.69
1_9	148,181,900	0.47	69.69
1_10	127,532,184	0.73	44.92
1_14	265,534,720	0.44	45.81
2_4	302,911,430	0.17	26.15
2_10	200,927,014	0.36	54.40
2_14	138,048,364	0.12	23.87
2_15	297,177,502	0.38	42.04
3_3	183,571,350	0.65	55.76
3_5	175,591,306	0.38	57.62
3_7	214,847,606	0.28	41.78
3_12	176,541,044	0.32	29.99
4_1	163,295,084	0.27	56.09
4_3	123,870,986	0.52	49.52
4_10	400,583,236	0.11	23.70
4_13	115,595,890	0.24	32.19

^1^ First symbol in the name represents weeks after inoculation.

**Table 2:** Unique GO terms (level 7) for the top 100 expressed transcripts at each time point.

Biological Process	Unique for Time Point
Histidine biosynthetic process	1 WPI
Arginine biosynthetic process	1 WPI
Coenzyme A metabolic process	1 WPI
Response to heat	1 WPI
Response to oxygen-containing compound	1 WPI
Isoprenoid biosynthetic process	1 WPI
Response to osmotic stress	1 WPI
Response to oxidative stress	1 WPI
Glyoxylate cycle	1 WPI
Carboxylic acid metabolic process	1 WPI
Protein-containing complex assembly	1 WPI
S-adenosylmethionine biosynthetic process	1 WPI
Proton transmembrane transport	2 WPI
Negative regulation of protein modification process	2 WPI
Negative regulation of phosphate metabolic process	2 WPI
DNA-templated transcription	2 WPI
Macroautophagy	2 WPI
Regulation of translation	2 WPI
Cellular response to amino acid starvation	2 WPI
Regulation of protein dephosphorylation	2 WPI
Positive regulation of transcription by RNA polymerase II	2 WPI
Acetyl-coa biosynthetic process	3 WPI
Citrate metabolic process	3 WPI
Cellular biosynthetic process	4 WPI
Protein import into mitochondrial matrix	4 WPI
Signal transduction	4 WPI
Ergosterol biosynthetic process	4 WPI

WPI—weeks post-inoculation.

**Table 3:** Twelve (ten annotated) most upregulated and six downregulated transcripts (1 WPI vs. 2 WPI).

Mapping Reference ID	Annotation	Fold Change	*p*-Value
Upregulated			
CCPA1999.b1	Hypothetical protein HETIRDRAFT_426980	181.85	4.72 × 10^−5^
CCPB2345.b1	Carotenoid ester lipase precursor	145.16	2.55 × 10^−4^
CCOZ2064.b1	ATP-utilizing phosphoenolpyruvate carboxykinase	64.23	9.74 × 10^−5^
CCPA2867.g1	Aldo/keto reductase	45.65	1.76 × 10^−4^
CCPB993.g1	Terpenoid cyclases/protein prenyltransferase alpha-alpha toroid	43.52	5.23 × 10^−3^
CCPC5739.g1	NAD-P-binding protein	34.14	1.76 × 10^−3^
CCPC2435.b1	Na *	33.93	2.87 × 10^−4^
CCPA4098.g1	GPI mannosyltransferase 3	29.59	2.71 × 10^−3^
CCPB1601.b1	Na	29.53	8.77 × 10^−4^
CCOZ1600.b1	Methionine adenosyltransferase	28.24	4.23 × 10^−3^
CCPC3360.b1	Isocitrate lyase	27.32	9.33 × 10^−5^
CCPA4929.b1	Alpha/beta hydrolase	25.73	5.18 × 10^−4^
Downregulated			
CCPC8078.b1	Transcription regulator	−23.45	7.99 × 10^−3^
CCOZ5192.g1	Dnaj domain-containing protein	−20.35	3.12 × 10^−3^
CCPB3914.b1	Hypothetical protein HETIRDRAFT_426907	−18.18	4.34 × 10^−3^
CCOZ3764.b1	Negative regulator of differentiation 1	−12.93	9.40 × 10^−3^
CCPC2832.b1	Ornithine decarboxylase antizyme domain-containing protein	−12.24	8.22 × 10^−3^
CCPA3492.b1	Hypothetical protein HETIRDRAFT_477666	−8.36	3.06 × 10^−3^

* Na—no annotation.

**Table 4:** Ten most upregulated and eleven (ten annotated) most downregulated transcripts (1 WPI vs. 3 WPI).

Mapping Reference ID	Annotation	Fold Change	*p*-Value
Upregulated			
CCPC2187.b1	Malic enzyme	61.69	2.06 × 10^−4^
CCOZ3444.b1	Heat shock protein 70	55.59	3.80 × 10^−3^
CCPB993.g1	Terpenoid cyclases/protein prenyltransferase alpha-alpha toroid	44.46	1.40 × 10^−3^
CCPC2829.b1	Pali domain-containing protein	32.82	2.45 × 10^−4^
CCPA4010.b1	Fatty acid desaturase domain-containing protein	27.64	4.38 × 10^−3^
CCPC4213.b1	Hypothetical protein HETIRDRAFT_325943	25.72	5.16 × 10^−3^
11E44-04-08	Predicted protein	24.54	8.11 × 10^−4^
CCPC4213.g1	Hypothetical protein HETIRDRAFT_325943	24.37	6.08 × 10^−3^
CCPA4569.b1	Hypothetical protein HETIRDRAFT_441917	23.75	3.45 × 10^−4^
CCPC6772.g1	Putative BAG domain-containing protein	23.30	7.21 × 10^−3^
Downregulated			
CCPC3479.g1	Groes-like protein	−41.47	1.64 × 10^−3^
CCPA5017.g1	Protein arginine N-methyltransferase	−39.95	3.01 × 10^−4^
CCOZ3601.b1	Secy protein	−27.45	6.26 × 10^−3^
16D10	HSP20-like chaperone	−25.79	2.78 × 10^−3^
CCOZ4082.b1	Glucoamylase	−23.26	2.19 × 10^−3^
CCPA3011.b1	Cell division control/GTP-binding protein	−20.94	8.14 × 10^−3^
CCPA3961.g1	Glutamate decarboxylase	−18.18	7.47 × 10^−3^
CCPC7984.b1	Na *	−17.48	5.70 × 10^−3^
D69E9	40S ribosomal protein S26	−16.73	3.76 × 10^−3^
CCPB4097.b1	Hypothetical protein HETIRDRAFT_439855	−14.76	4.80 × 10^−3^
CCPC6286.b1	Leucine aminopeptidase	−13.02	7.79 × 10^−3^

* Na—no annotation.

**Table 5:** Ten most upregulated and seven downregulated transcripts (1 WPI vs. 4 WPI).

Mapping Reference ID	Annotation	Fold Change	*p*-Value
Upregulated			
CCPA575.b1	Delta-12 fatty acid desaturase	48.12	7.94 × 10^−5^
CCPA1686.b1	Polysaccharide lyase family 1 protein	47.55	4.24 × 10^−3^
CCPA1999.b1	Hypothetical protein HETIRDRAFT_426980	46.41	1.60 × 10^−4^
10F24-03-16	Elongase of fatty acids ELO	45.14	9.47 × 10^−3^
CCPC993.b1	Erylysin B	38.75	5.07 × 10^−3^
CCPC1268.b1	Delta-12 fatty acid desaturase protein	38.60	1.89 × 10^−3^
CCPA3999.b1	Fatty acid desaturase domain-containing protein	35.32	4.51 × 10^−3^
CCPC5436.b1	Hypothetical protein EW146_g3762	33.84	4.25 × 10^−3^
CCPA5235.g1	Hypothetical protein HETIRDRAFT_468348	33.44	3.52 × 10^−3^
CCPC8046.b1	Delta-12 fatty acid desaturase	33.02	1.59 × 10^−4^
Downregulated			
D128H9	RS27A protein	−128.69	2.57 × 10^−4^
CCPA2234.b1	Hypothetical protein HETIRDRAFT_409605	−50.89	7.84 × 10^−4^
CCOZ3606.b1	Glycoside hydrolase superfamily	−21.73	5.62 × 10^−3^
CCPB3914.b1	Hypothetical protein HETIRDRAFT_426907	−20.09	3.36 × 10^−3^
CCPA5017.g1	Protein arginine N-methyltransferase	−18.89	5.61 × 10^−3^
CCPA2400.b1	General substrate transporter	−13.93	8.68 × 10^−3^
CCPB4930.b1	Family 43 glycosylhydrolase	−11.52	3.40 × 10^−3^

## Data Availability

The transcriptome sequencing reads have been deposited into the NCBI SRA archive with the BioProject ID PRJNA985902 and accession numbers SRR25032047–SRR25032062 for the individual sequencing libraries.

## Supplementary Materials

The following supporting information can be downloaded at https://www.mdpi.com/article/10.3390/ijms252111375/s1.
