# Supplementary material for: Early-Stage Infection-Specific Heterobasidion annosum (Fr.) Bref. Transcripts in H. annosum–Pinus sylvestris L. Pathosystem
Source: Int J Mol Sci. 2024 Oct 23;25(21):11375. doi: 10.3390/ijms252111375 (PMC11546620; doi:10.3390/ijms252111375)
Supplement: Supplementary file 1 [file ijms-25-11375-s001.zip › Supplementary File S2 - Most up- and downregulated transcripts for DGE comparisons 2 WPI vs 3 WPI, 2 WPI vs 4 WPI and 3 WPI vs 4 WPI.pdf]

Eight upregulated and ten most downregulated transcripts (2 WPI vs 3WPI)

Mapping Reference ID	Annotation	Fold change	P-value
	Upregulated		
CCPB3963.b1	Pyruvate/Phosphoenolpyruvate kinase-like domain-containing protein	32.61	7.45E-03
CCPC2829.b1	Pali-domain-containing protein	29.64	4.15E-04
CCPA3943.g1	Hypothetical protein EW146_g8910	24.96	3.93E-03
CCPB3344.g1	Alpha,alpha-trehalase-neutral trehalase	24.01	2.82E-03
CCPA416.g1	Hypothetical protein HETIRDRAFT_423383	18.16	2.56E-03
CCPB765.b1	Prolyl oligopeptidase	14.65	6.22E-03
CCOZ3764.b1	Negative regulator of differentiation 1	14.24	9.70E-03
CCOZ5196.g1	Hypothetical protein HETIRDRAFT_432684	10.79	8.32E-03
	Downregulated		
CCPA2867.g1	Aldo/keto reductase	–60.69	5.54E-05
CCPA1999.b1	Hypothetical protein HETIRDRAFT_426980	–49.97	2.30E-03
CCPB1903.g1	Ubiquitin-related domain-containing protein	–48.89	3.26E-03
CCPA586.b1	Metalloprotease	–46.33	4.96E-04
CCPC4609.g1	Hypothetical protein HETIRDRAFT_479197	–45.19	1.25E-03
CCOZ2064.b1	ATP-utilizing phosphoenolpyruvate carboxykinase	–29.08	1.66E-03
CCOZ1602.b1	Heat shock protein 70	–28.80	2.14E-03
CCPB3190.g1	Acid protease	–27.19	3.77E-04
CCPA3562.b1	Fungalysin metallopeptidase-domain-containing protein	–27.04	1.85E-03
CCOZ1600.b1	Methionine adenosyltransferase	–26.79	4.95E-03

Three upregulated and four (three annotated) downregulated transcripts (2 WPI vs 4WPI)

Mapping Reference ID	Annotation	Fold change	P-value
	Upregulated		
CCPA1686.b1	Polysaccharide lyase family 1 protein	48.537	4.07E-03
CCPA416.g1	Hypothetical protein HETIRDRAFT_423383	14.1509	7.95E-03
CCOZ4816.g1	Cyclin-domain-containing protein	13.1396	6.60E-03
	Downregulated		
CCPA2867.g1	Aldo/keto reductase	–29.01	1.03E-03
CCPB1601.b1	---Na---	–25.35	1.67E-03
CCPB3190.g1	Acid protease	–12.75	7.47E-03
CCPC2967.g1	Hypothetical protein HETIRDRAFT_388079	–11.20	4.76E-03

Four upregulated and three downregulated transcripts (3 WPI vs 4WPI)

Mapping Reference ID	Annotation	Fold change	P-value
	Upregulated		
CCPA3562.b1	Fungalysin metallopeptidase-domain-containing protein	15.00	9.48E-03
CCOZ1823.b1	Fungalysin metallopeptidase-domain-containing protein	27.98	2.95E-03
CCOZ616.b1	Class III ADH enzyme	31.86	8.39E-03
CCPC3198.g1	Hypothetical protein HETIRDRAFT_437586	32.83	1.21E-03
	Downregulated		
CCPC3638.b1	Ribosomal protein S2, flavodoxin-like domain-containing protein	–18.68	5.52E-03
CCPC4426.b1	Peptidase C15 pyroglutamyl peptidase I-like protein	–15.80	8.20E-03
CCOZ5196.g1	Hypothetical protein HETIRDRAFT_432684	–11.42	7.49E-03
