# Supplementary figures and images for: Early-Stage Infection-Specific Heterobasidion annosum (Fr.) Bref. Transcripts in H. annosum–Pinus sylvestris L. Pathosystem
Source: Int J Mol Sci. 2024 Oct 23;25(21):11375. doi: 10.3390/ijms252111375 (PMC11546620; doi:10.3390/ijms252111375)

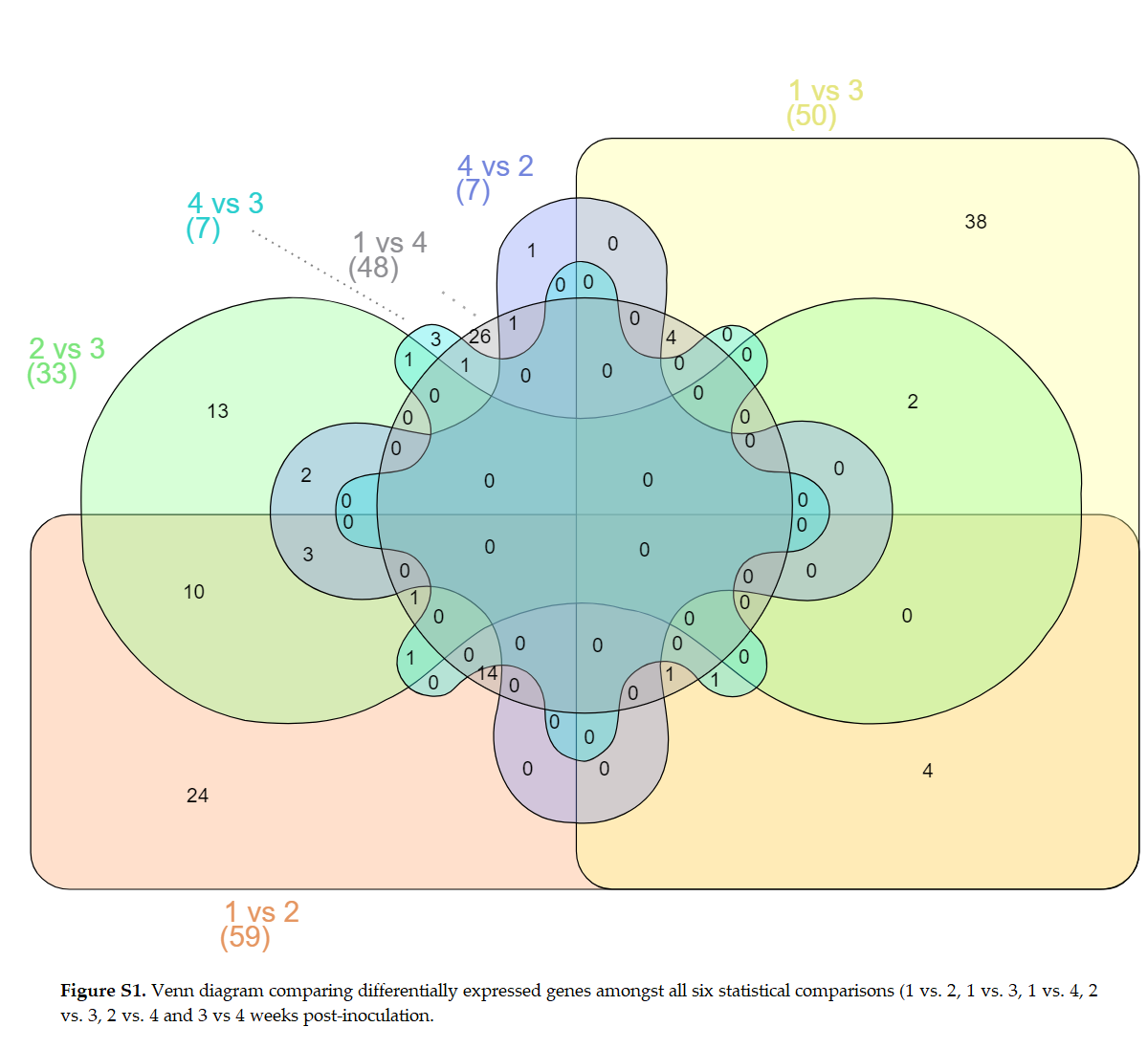

Supplement: Supplementary file 1 [file ijms-25-11375-s001.zip › Supplementary Figure S1 - Venn diagram of all compared timepoints.png]
